# Biomarkers from distinct biological pathways improve early risk stratification in medical emergency patients: the multinational, prospective, observational TRIAGE study

**DOI:** 10.1186/s13054-015-1098-z

**Published:** 2015-10-29

**Authors:** Philipp Schuetz, Pierre Hausfater, Devendra Amin, Adina Amin, Sebastian Haubitz, Lukas Faessler, Alexander Kutz, Antoinette Conca, Barbara Reutlinger, Pauline Canavaggio, Gabrielle Sauvin, Maguy Bernard, Andreas Huber, Beat Mueller, for the TRIAGE Study group

**Affiliations:** Division of General and Emergency Medicine, University Department of Medicine, Kantonsspital Aarau, Tellstrasse, 5001 Aarau, Switzerland; Emergency Department, Groupe Hospitalier Pitié-Salpêtrière Assistance Publique-Hôpitaux de Paris (APHP), Paris, France; Department of critical care, Morton Plant Hospital, 300 Pinellas Street, Clearwater, FL 33756 USA; Department of Clinical Nursing Science, Kantonsspital Aarau, Tellstrasse, 5001 Aarau, Switzerland; Department of Laboratory Medicine, Kantonsspital Aarau, Tellstrasse, 5001 Aarau, Switzerland; Medical Faculty of the University of Basel, Basel, Switzerland; Biochemistry Department, Hôpital Pitié-Salpêtrière and Univ-Paris Descartes, Paris, France

## Abstract

**Introduction:**

Early risk stratification in the emergency department (ED) is vital to reduce time to effective treatment in high-risk patients and to improve patient flow. Yet, there is a lack of investigations evaluating the incremental usefulness of multiple biomarkers measured upon admission from distinct biological pathways for predicting fatal outcome and high initial treatment urgency in unselected ED patients in a multicenter and multinational setting.

**Method:**

We included consecutive, adult, medical patients seeking ED care into this observational, cohort study in Switzerland, France and the USA. We recorded initial clinical parameters and batch-measured prognostic biomarkers of inflammation (pro-adrenomedullin [ProADM]), stress (copeptin) and infection (procalcitonin).

**Results:**

During a 30-day follow-up, 331 of 7132 (4.6 %) participants reached the primary endpoint of death within 30 days. In logistic regression models adjusted for conventional risk factors available at ED admission, all three biomarkers strongly predicted the risk of death (AUC 0.83, 0.78 and 0.75), ICU admission (AUC 0.67, 0.69 and 0.62) and high initial triage priority (0.67, 0.66 and 0.58). For the prediction of death, ProADM significantly improved regression models including (a) clinical information available at ED admission (AUC increase from 0.79 to 0.84), (b) full clinical information at ED discharge (AUC increase from 0.85 to 0.88), and (c) triage information (AUC increase from 0.67 to 0.83) (*p* <0.01 for each comparison). Similarly, ProADM also improved clinical models for prediction of ICU admission and high initial treatment urgency. Results were robust in regard to predefined patient subgroups by center, main diagnosis, presenting symptoms, age and gender.

**Conclusions:**

Combination of clinical information with results of blood biomarkers measured upon ED admission allows early and more adequate risk stratification in individual unselected medical ED patients. A randomized trial is needed to answer the question whether biomarker-guided initial patient triage reduces time to initial treatment of high-risk patients in the ED and thereby improves patient flow and clinical outcomes.

**Trial registration:**

ClinicalTrials.gov NCT01768494. Registered January 9, 2013.

## Introduction

Emergency departments (ED) are increasingly crowded by patients with both urgent and nonurgent health issues [1, 2]. As a consequence, patients needing urgent care may not be treated in time resulting in poor health outcomes [3, 4]. Earlier risk stratification has the potential to reduce time to effective treatment - a main predictor for patient outcome across different medical conditions, including septicemia [5], pneumonia [6], stroke (“time is brain”) [7], and myocardial infarction (“time is heart”) [8]. Risk stratification may also improve patient flow and initial site-of-care decisions (i.e., outpatient versus inpatient management). Hence, international guidelines recommend the use of risk scores in well-defined patient populations such as the Pneumonia Severity Index (PSI) in community-acquired pneumonia (CAP) [9]. Yet, there is a lack of a more general risk stratification score for undifferentiated medical patients at the most proximal time point of seeking ED care. At this earliest time point in ED care, risk stratification is most challenging due to lack of thorough clinical information, but may have the biggest potential to reduce time to effective treatment of patients and to improve patient flow.

Several triage systems have been proposed including the Manchester Triage System (MTS), the Australasian Triage Scale (ATS), the Canadian Triage and Acuity Scale (CTAS) and the Emergency Severity Index (ESI) [10, 11]. These scores assign patients based on their presenting symptoms and a combination of vital signs into risk categories with different recommended times for physician assessment [10]. The main rational of these score is to stratify treatment urgency based on clinical “red flags”. Yet, only few rigorous clinical studies have investigated the performance of initial triage scores for their ability to improve initial triage decisions and for outcome prediction [10].

In addition to triage scores, there is high potential in the use of prognostic biomarkers from distinct biological pathways to identify persons who are at risk for high treatment urgency and adverse medical outcome [12]. Many biomarkers have been related to risk in selected ED patient populations, including the prognostic inflammatory marker pro-adrenomedullin (ProADM) [13–19], the stress marker pro-vasopressin (copeptin) [19–23] and the bacterial infection marker procalcitonin (PCT) [24–27]. Measurement of these biomarkers simultaneously (a “multimarker” approach) in unselected ED patients could enhance early risk stratification.

Herein, we evaluated the incremental usefulness of these three previously reported prognostic biomarkers from distinct biological pathways in a large, international ED patient cohort for predicting death, admission to the intensive care unit (ICU) and high treatment urgency where delays in initial treatment may have detrimental consequences.

## Methods

### Study design

The TRIAGE study is a multinational, prospective, observational cohort study. From March 2013 to October 2014, we included consecutive medical patients presenting with a medical urgency at three tertiary-care hospitals in Aarau (Switzerland), Paris (France) or Clearwater (FL, USA), respectively. The Swiss hospital (Kantonsspital Aarau) is a 600-bed tertiary-care hospital with most medical admissions entering the hospital over the ED. The French hospital (Hôpital Pitié-Salpêtrière, Paris) is a large inner-city 1800-bed referral center. The US hospital (Morton Plant Hospital, Clearwater, FL) is a 687-bed community referral center. As an observational quality control study, the Institutional Review Boards (IRB) of the three hospitals approved the study and waived the need for individual informed consent (main Swiss IRB: Ethikkommission Kanton Aargau (EK 2012/059); French IRB: CCTIRS - Comité consultatif sur le traitement de l’information en matière de recherche (C.C.T.I.R.S.), (CPP ID RCB: 2013-A00129-36); US IRB MPM-SAH Institutional Review Board, Clearwater FL [IRB number 2013_005]). The study was registered at the “ClinicalTrials.gov” registration website [28] and the study protocol has been published previously [29].

### Patient sample

Patients seeking ED care for medical health issues and meeting our inclusion criteria at one of the participating hospital EDs were consecutively included. Inclusion criteria were adult medical patients in whom an initial blood draw was done as part of the routine ED assessment. We defined “medical patient” as a patient with an initial predominant medical health issue as judged by the triage nurse. We excluded surgical and pediatric patients, but had no other exclusion criteria in regard to main diagnosis or presenting symptoms to reflect the diversity and challenges of “real life”.

### Data collection

Upon ED admission, all patients were assessed by a triage nurse and initial triage priority was assigned based on the routine hospital algorithm [10]. All participating centers categorize patients upon ED admission into triage class using a triage score as part of their routine clinical care. The Swiss center uses the German version of the five-level MTS, which has been validated previously [30]. The US center uses the five-level Morton Plant Hospital Triage Acuity Scale based on the French center uses a similar five-level scoring system, which has also been validated in France [31]. All participants provided a medical history and underwent a physical examination with measurement of vital signs and laboratory assessment with collection of leftover blood samples. We also recorded the main presenting clinical symptoms and complaints, sociodemographics and comorbidities. Upon ED discharge, two independent attending ED physicians adjudicated a medical triage priority based on all medical results available at this time to all patients (high versus low triage priority). In case of disagreement, the case was discussed with a third independent physician until consensus was reached. All information was entered into a case report form (CRF) and stored in a centralized, password-secured databank (SecuTrial).

Throughout the hospital stay, physicians, nurses and social care workers managed patients in accordance to hospital guidelines according to the underlying medical condition and independent of the research team. All patients were contacted 30 days after hospital admission for a telephone interview with a predefined questionnaire to assess vital and functional status, hospital readmission, as well as quality of life, care needs at home and satisfaction with care provided.

### Overall hypothesis and research aim

The overall hypothesis of this study is that the addition of prognostic blood markers from distinct biological pathways will improve initial clinical parameters and vital signs for risk stratification and initial triage of patients at an early stage of ED admission. In turn, this may translate into better patient flow and more adequate estimation of triage priority and site-of-care decisions.

### Primary and secondary endpoints

One primary and two secondary endpoints were predefined for inclusion in the main prediction analysis. The primary endpoint was defined as all-cause 30-day mortality. To assess vital status, we followed all patients throughout the hospital stay and contacted them by telephone interview 30 days after admission. In case a patient could not be reached, we contacted the family or the patient’s general practitioner to assess vital status.

Secondary endpoints were admission to the ICU within 30 days following ED admission and high initial adjudicated triage priority. The decision for ICU admission was left to the discretion of the treating physicians. As outlined above, initial high triage priority was adjudicated by two independent ED physicians. Similar to a previous study, the physicians evaluated post hoc what the real degree of urgency (the “gold standard”) would have been, based on all available ED data, results of diagnostic tests, and the final diagnosis at ED discharge. Specifically, the main question for the adjudicators was “*under difficult circumstances, what is the maximum possible time that this patient would have been able to wait before being seen?*” with options of “patient could not wait”, 10 minutes, 30 minutes, 90 minutes, or “no emergency”. To further standardize the adjudication, we developed examples prior to the start of this study, which were distributed to adjudicators. In case of disagreement, a third independent physician reviewed the case until consensus was reached among the three. We then dichotomized the initial five priority categories into two categories (i.e., low priority [more than 10 minutes, class 3, 4 or 5] versus high priority [less than 10 minutes, class 1 or 2]).

### Definitions of diagnoses and main symptoms at ED admission

Based on the information available at ED discharge, patients were grouped into different main diagnoses (infection, gastrointestinal disease, cardiovascular disease, worsening of general condition, neurological disease, other disease) and symptom categories (general malaise, pain, gastrointestinal symptom, neurological symptom, fever, respiratory symptom). Thereby, the group of “neurological symptoms” included reduced consciousness, dizziness, confusion, syncope or the state shortly after, and neurological deficits.

### Blood draws and candidate biomarkers

Leftover blood samples of routinely collect blood tubes on admission were immediately centrifuged, aliquoted and frozen at −20 °C for later batch analysis of blood biomarkers. The results of these analyses were not available at the time of hospitalization of the patients and, thus, physicians, patients and outcome adjudicators were blinded to their results. We decided to examine blood markers from three different distinct biologic pathways as candidate biomarkers, namely inflammation (ProADM) [13–19], stress (copeptin) [19–23] and bacterial infection (PCT) [24–27] based on the previous literature demonstrating a high prognostic potential of these markers. ProADM and copeptin were batch-measured in plasma with a new sandwich immunoassay as described elsewhere [32–34]. The assays have analytical detection limits of 0.08 nmol/L and 0.4 pmol/L, respectively. PCT was measured with a highly sensitive time-resolved amplified cryptate emission (TRACE) technology assay (PCT Kryptor®, B.R.A.H.M.S. AG, Hennigsdorf, Germany). The assay has a detection limit of 0.02 ug/L and functional assay sensitivity of 0.06 μg/L.

### Statistical considerations and sample size

We aimed to include a total of at least 7000 patients with expected rates for mortality of 5 % (*n* = 350), ICU admission of 7 % (*n* = 490) and high treatment priority of 40 % (*n* = 2800). This provides 35–280 degrees of freedom for each model (with ten outcome in the data set per degree of freedom in the statistical model), and thus high power for the calculation of the main multivariate models overall and in predefined subgroups.

We used multivariable logistic regression models to examine the association of biomarker levels with the primary and the two secondary endpoints. The distribution of raw biomarker data was skewed. After log transformation with a base of ten, the distribution of the biomarker data approximated a normal distribution. We report odds ratios (ORs) and 95 % confidence intervals (CIs) as a measure of association and C-statistics (area under the curve [AUC]) as a measure of discrimination. ORs thereby correspond to a one-unit increase in the explanatory variable and to any tenfold increase in log-transformed biomarker levels (log transformed with a base of ten). As predefined, regression models were adjusted for age and gender (model 1), age, gender, comorbidities, main symptom, main diagnosis (model 2), and for age, gender, comorbidities, main symptom, main diagnosis, vital signs (model 3). To assess the incremental usefulness of one or all biomarker levels to the clinical information available at ED admission, we performed a head-to-head comparison between statistical models limited to clinical information and models with clinical and biomarker information. For this analysis we predefined the clinical models as “initial clinical model” (including only information available at ED admission such as age, gender, main presenting symptom and vital signs), “full clinical model” (including all clinical parameters available at ED discharge such as age, gender, comorbidities, main presenting symptom, main diagnosis and vital signs); and “triage scores models” (including the triage score (one to five triage classes) as assessed in the emergency department). For nested model comparisons, we used the likelihood ratio test as recommended [35]. Finally, we also investigated subgroups for differences in performance between centers, main diagnoses, main symptoms and sociodemographic factors (age).

The effects of adding ProADM to clinical information were further assessed using reclassification methods [36]. The analyses used continuous variable information with evaluation of the effects on risk category reclassification for all three endpoints. This approach separately analyzed the reclassification of persons who did or did not reach the outcome of interest. Reclassification to a higher risk group was considered upward movement in classification for participants not reaching the endpoint. On the other hand, reclassification downward was considered a failure for participants reaching the endpoint. We used risk categories of 5 and 10 % for mortality and ICU admission, and 20 and 50 % for triage priority. We calculated the net reclassification improvement (NRI), which assesses improvement in reclassification over risk categories; we also assessed integrated discrimination improvement (IDI), which can be viewed as a continuous version of the NRI without the recourse to a priori defined risk categories.

Discrete variables are expressed as frequency (percentage) and continuous variables as means and standard deviations (SDs) or medians and interquartile ranges (IQRs). Analyses were performed with STATA 12.2 (StataCorp, College Station, TX, USA).

## Results

### Patient population

A total of 7342 patients were included in the ED of the participating Swiss, US and French hospitals of whom 7132 patients had follow-up information and were included in the final analysis (*n* = 4579, *n* = 1000 and *n* = 1553). A detailed flow diagram is displayed in the Appendix. The median age was 62 years and 46.7 % of patients were females. The most frequent main complaints at ED admission were neurological symptoms (19.3 %), nonthoracic (17.1 %) and thoracic (14.6 %) pain and respiratory symptoms (13.3 %). The most prevalent main diagnoses were cardiovascular diseases (24.3 %), neurological diseases (22.0 %) and infections (12.6 %). Patients had a high burden of comorbidities including hypertension (39.2 %), coronary heart disease (11.7 %). diabetes (15.3 %) and cancer (13.6 %). Most patients were treated as inpatients (72.8 %) with a mean length of stay (LOS) of 6.0 days and variability among trial sites (range 38.4–100 %). Overall, 46.0 % of patients were adjudicated as having a high initial treatment priority. In regard to adverse outcome within 30 days of ED admission, 6.4 % of patients were admitted to ICU, 2.6 % died in the hospital and 4.6 % died within 30 day of admission. Additional baseline and follow-up patient characteristics of the overall population and stratified according to trial site are presented in Table 1.

**Table 1:** Patient characteristics overall and according to study site

	All patients	Swiss	US	French
*n* =	7132	4579	1000	1553
Sociodemographics				
Age, median (IQR)	62 (46, 76)	63 (47, 76)	69 (55, 81)	55 (38, 69)
Female gender, *n* (%)	3301 (46.7 %)	2046 (44.7 %)	500 (50.0 %)	755 (50.7 %)
Clinical presentation at ED admission, median (IQR)				
Blood pressure diastolic (mmHg)	80 (70, 90)	81 (71, 91)	77.5 (67, 89)	79 (70, 90)
Blood pressure systolic (mmHg)	137 (121, 154)	138 (122, 155)	141 (121, 163)	133 (119, 149)
Confusion, *n* (%)	522 (7.3 %)	453 (9.9 %)	40 (4.0 %)	29 (1.9 %)
Pulse (bpm)	83 (71, 97)	82 (70, 95)	82 (71, 98)	86 (74, 99)
Respiratory rate (breaths/min)	18 (18, 20)	32 (24, 36)	18 (18, 20)	21 (18, 25)
SpO2 (%)	96.8 (94, 98)	96 (94, 97)	97 (95, 99)	98 (96, 99)
Temperature (°C),	36.8 (36.4, 37.2)	36.9 (36.6, 37.4)	36.5 (36.1, 36.8)	36.7 (36.4, 37)
Main symptom at ED admission, *n* (%)				
Diarrhea, vomitus, dysuria	495 (6.9 %)	288 (6.3 %)	95 (9.5 %)	112 (7.2 %)
Fever	343 (4.8 %)	254 (5.5 %)	15 (1.5 %)	74 (4.8 %)
Gastrointestinal bleeding	199 (2.8 %)	140 (3.1 %)	28 (2.8 %)	31 (2.0 %)
Neurological symptoms	1379 (19.3 %)	1138 (24.9 %)	78 (7.8 %)	163 (10.5 %)
Nonthoracic pain	1217 (17.1 %)	682 (14.9 %)	103 (10.3 %)	432 (27.8 %)
Respiratory symptoms	948 (13.3 %)	526 (11.5 %)	220 (22.0 %)	202 (13.0 %)
Thoracic pain	1038 (14.6 %)	690 (15.1 %)	153 (15.3 %)	195 (12.6 %)
Worsening of general condition	837 (11.7 %)	258 (5.6 %)	308 (30.8 %)	271 (17.5 %)
Other symptom	676 (9.5 %)	603 (13.2 %)	0 (0.0 %)	73 (4.7 %)
Main diagnosis, *n* (%)				
Cancer	297 (4.2 %)	234 (5.1 %)	29 (2.9 %)	34 (2.2 %)
Cardiovascular	1732 (24.3 %)	1003 (21.9 %)	413 (41.3 %)	316 (20.3 %)
Gastrointestinal	896 (12.6 %)	503 (11.0 %)	92 (9.2 %)	301 (19.4 %)
Infection	896 (12.6 %)	657 (14.3 %)	49 (4.9 %)	190 (12.2 %)
Metabolic	238 (3.3 %)	62 (1.4 %)	56 (5.6 %)	120 (7.7 %)
Neurological	1569 (22.0 %)	1150 (25.1 %)	160 (16.0 %)	259 (16.7 %)
Pulmonary	471 (6.6 %)	169 (3.7 %)	158 (15.8 %)	144 (9.3 %)
Other	1033 (14.5 %)	801 (17.5 %)	43 (4.3 %)	189 (12.2 %)
Comorbidities, *n* (%)				
Cancer	968 (13.6 %)	678 (14.8 %)	79 (7.9 %)	211 (13.6 %)
Chronic renal failure	872 (12.2 %)	694 (15.2 %)	78 (7.8 %)	100 (6.4 %)
Congestive heart failure	487 (6.8 %)	286 (6.2 %)	118 (11.8 %)	83 (5.3 %)
COPD	359 (5.0 %)	229 (5.0 %)	51 (5.1 %)	79 (5.1 %)
Coronary heart disease	838 (11.7 %)	557 (12.2 %)	107 (10.7 %)	174 (11.2 %)
Dementia	223 (3.1 %)	147 (3.2 %)	67 (6.7 %)	9 (0.6 %)
Diabetes	1088 (15.3 %)	681 (14.9 %)	224 (22.4 %)	183 (11.8 %)
History of stroke	566 (7.9 %)	456 (10.0 %)	5 (0.5 %)	105 (6.8 %)
Hypertension	2795 (39.2 %)	1920 (41.9 %)	479 (47.9 %)	396 (25.5 %)
Substance abuse	460 (6.4 %)	303 (6.6 %)	52 (5.2 %)	105 (6.8 %)
Initial blood biomarkers, median (IQR)				
Copeptin, (pmol/L)	10.74 (4.54, 38.90)	11.60 (4.70, 43.80)	15.02 (5.70, 59.42)	7.63 (3.95, 19.21)
Creatinine (micromol/L)	81.0 (67.0, 103.0)	85.0 (70.0, 106.0)	79.6 (70.7, 106.1)	67.0 (55.0, 84.0)
Glucose (mmol/L)	6.1 (5.3, 7.5)	6.2 (5.4, 7.5)	6.3 (5.3, 8.2)	5.8 (5.2, 6.9)
Hemoglobin (G/L)	13.6 (12.1, 14.8)	13.7 (12.2, 14.9)	12.9 (11.4, 14.4)	13.7 (12.4, 14.8)
PCT (ug/L)	0.08 (0.06, 0.13)	0.08 (0.06, 0.13)	0.09 (0.06, 0.15)	0.08 (0.06, 0.13)
ProADM (nmol/L)	0.79 (0.57, 1.24)	0.81 (0.60, 1.27)	0.98 (0.70, 1.62)	0.59 (0.45, 0.88)
Sodium (mmol/L)	139 (137, 140)	139 (137, 141)	138 (136, 140)	138 (137, 140)
WBC (G/L)	8.4 (6.6, 10.9)	8.5 (6.7, 11.0)	8.3 (6.4, 11)	8.1 (6.3, 10.8)
Initial triage score, *n* (%)				
No emergency	774 (11.1 %)	638 (14.2 %)	0 (0.0 %)	136 (9.1 %)
Within 90 minutes	1746 (25.1 %)	1030 (23.0 %)	61 (6.1 %)	655 (43.9 %)
Within 30 minutes	3030 (43.5 %)	1783 (39.8 %)	693 (69.3 %)	554 (37.2 %)
Within 10 minutes	1256 (18.0 %)	910 (20.3 %)	219 (21.9 %)	127 (8.5 %)
Immediate treatment needed	163 (2.3 %)	117 (2.6 %)	27 (2.7 %)	19 (1.3 %)
Patient outcomes, *n* (%)				
Site of care, *n* (%)				
Outpatient treatment	1555 (27.2 %)	637 (19.8 %)	0 (0.0 %)	918 (61.6 %)
Length of stay, mean (SD)	6.0 (6.1)	5.7 (5.1)	5.0 (5.3)	9.5 (9.5)
Location after hospital/ED discharge, *n* (%)				
Home	5983 (83.9 %)	3796 (82.9 %)	723 (72.3 %)	1464 (94.3 %)
Other institution	504 (7.1 %)	435 (9.5 %)	61 (6.1 %)	8 (0.5 %)
Rehabilitation	320 (4.5 %)	207 (4.5 %)	50 (5.0 %)	63 (4.1 %)
Other	325 (4.6 %)	141 (3.1 %)	166 (16.6 %)	18 (1.2 %)
Outcomes within 30 days, *n* (%)				
Intensive care unit admission	453 (6.4 %)	181 (4.0 %)	153 (15.3 %)	119 (7.7 %)
Inhospital mortality	188 (2.6 %)	136 (3.0 %)	21 (2.1 %)	31 (2.0 %)
30-day mortality	331 (4.6 %)	252 (5.5 %)	45 (4.5 %)	34 (2.2 %)
Unplanned hospital readmission	590 (8.3 %)	311 (6.8 %)	184 (18.4 %)	95 (6.1 %)

*IQR* interquartile range, *ED* emergency department, *COPD* chronic obstructive pulmonary disease, *PCT* procalcitonin, *ProADM* pro-adrenomedullin, *WBC* white blood cell count, *SD* standard deviation

### Association of initial blood markers and adverse outcome

In unadjusted logistic regression analysis, we found strong associations of all three measured biomarkers (ProADM, copeptin, PCT) and primary and secondary endpoints, namely 30-day mortality, admission to ICU and high initial treatment priority. These associations remained robust in different multivariate models including age and gender (model 1), age, gender, comorbidities, main symptom, main diagnosis (model 2), and age, gender, comorbidities, main symptom, main diagnosis, vital signs (model 3) (Table 2). For mortality prediction, ProADM had the best discrimination value with an AUC of 0.83 compared to 0.78 for copeptin and 0.75 for PCT. For prediction of ICU admission and high initial triage priority, ProADM and copeptin had the best discrimination values as compared to PCT (an AUC of ProADM 0.67 and 0.67, copeptin 0.69 and 0.66, PCT 0.62 vs. 0.58, respectively).

**Table 2:** Association of biomarkers with primary and secondary outcomes

	ProADM (nmol/L)	Copeptin (pmol/L)	Procalcitonin (ug/L)
30-day mortality			
Survivors, median (IQR)	0.8 (0.6 to 1.2)	10 (4 to 35)	0.08 (0.05 to 0.13)
Nonsurvivors, median (IQR)	1.8 (1.1 to 3.9)	62 (22 to 154)	0.17 (0.09 to 0.50)
Regression analysis, OR (95 % CI)			
Unadjusted model	32.3 (23.1, 45.2)	4.3 (3.6, 5.1)	2.7 (2.4, 3.2)
Multivariate model 1	20.4 (14.1, 29.4)	3.4 (2.8, 4)	2.5 (2.1, 2.9)
Multivariate model 2	22.4 (14.4, 34.9)	3.5 (2.8, 4.3)	2.5 (2.1, 3)
Multivariate model 3	21.4 (13.5, 34)	3.2 (2.6, 4)	2.4 (2, 2.9)
Discrimination statistics			
AUC (95 % CI)	0.83 (0.81, 0.85)	0.78 (0.75, 0.80)	0.75 (0.72, 0.77)
Admission to ICU			
No admission to ICU, median (IQR)	0.8 (0.6 to 1.2)	10 (5 to 35)	0.08 (0.06 to 0.13)
Admission to ICU, median (IQR)	1.2 (0.7 to 2.3)	41 (10 to 138)	0.10 (0.07 to 0.26)
Regression analysis, OR (95 % CI)			
Unadjusted model	7.7 (5.8, 10.3)	3 (2.6, 3.4)	1.9 (1.6, 2.1)
Multivariate model 1	8 (5.8, 11)	2.9 (2.5, 3.4)	1.7 (1.5, 2)
Multivariate model 2	10 (6.9, 14.5)	3 (2.6, 3.6)	2.1 (1.8, 2.5)
Multivariate model 3	7.7 (5.2, 11.5)	2.8 (2.4, 3.3)	1.9 (1.6, 2.3)
Discrimination statistics			
AUC (95 % CI)	0.67 (0.65, 0.70)	0.69 (0.67, 0.72)	0.62 (0.59, 0.64)
High initial triage priority			
Low triage priority, median (IQR)	0.7 (0.5 to 1.0)	8 (4 to 21)	0.07 (0.06 to 0.11)
High triage priority, median (IQR)	1.0 (0.7 to 1.6)	18 (6 to 68)	0.08 (0.06 to 0.16)
Regression analysis, OR (95 % CI)			
Unadjusted model	10 (8.2, 12.2)	2.6 (2.4, 2.8)	1.9 (1.7, 2.1)
Multivariate model 1	8.6 (6.8, 10.8)	2.2 (2, 2.4)	1.6 (1.5, 1.8)
Multivariate model 2	11.6 (8.8, 15.2)	2.3 (2.1, 2.6)	1.7 (1.5, 1.9)
Multivariate model 3	10.8 (8.2, 14.3)	2.2 (2, 2.5)	1.6 (1.4, 1.8)
Discrimination statistics			
AUC (95 % CI)	0.67 (0.66, 0.68)	0.66 (0.65, 0.67)	0.58 (0.56, 0.59)

Biomarker data were log transformed before entering into the statistical models with a base of ten. The ORs therefore correspond to a tenfold increase in biomarker levels. Model 1 (age, gender), model 2 (age, gender, comorbidities, main symptom, main diagnosis), model 3 (age, gender, comorbidities, main symptom, main diagnosis, vital signs)

*ProADM* pro-adrenomedullin, *IQR* interquartile range, *OR* odds ratio, *CI* confidence interval, *AUC* area under the curve

### Incremental value of ProADM and biomarker panel on clinical information

**Fig. 1:**
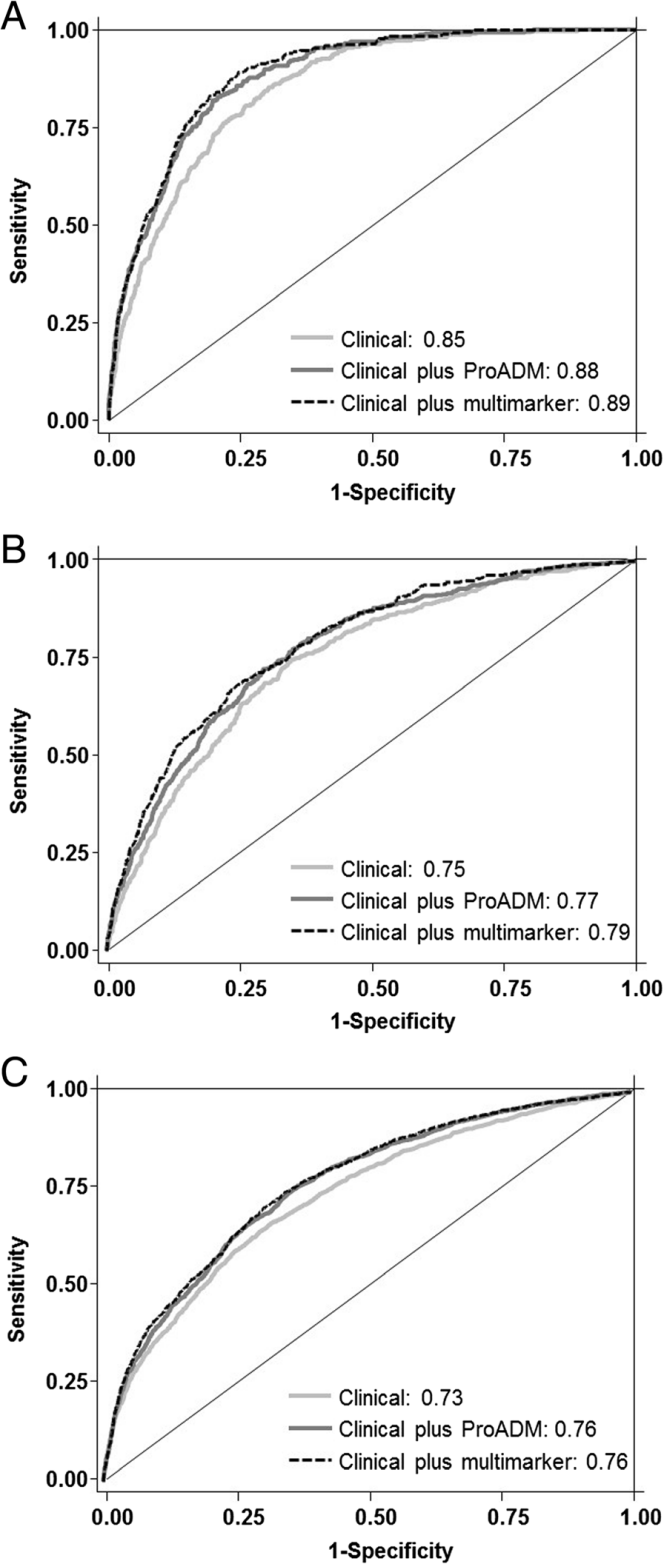
Receiver operating characteristic (ROC) analysis comparing three models for prediction of outcomes: a clinical model (**a**), a biomarker model (**b**) and a clinical plus biomarker model (**c**)

To study whether ProADM or a multimarker panel including all three biomarkers would improve risk prediction of available clinical information, we calculated different regression models based on (a) clinical information readily available at ED admission (age, gender, main presenting symptom and vital signs), (b) full clinical information available at ED discharge (age, gender, comorbidities, main presenting symptom, main diagnosis and vital signs) and (c) information from initial triage scores (Table 3 and Fig. 1). ProADM was found to substantially improve all models with best improvements for prediction of 30-day mortality (from AUC 0.79 to 0.84 for the model including clinical information readily available at ED admission; from AUC 0.85 to 0.88 for the model including full clinical information available at ED discharge; from AUC 0.67 to 0.83 for the model including information from initial triage scores. Compared to the model including ProADM only, the multimarker panel (including all three markers) provided only minimal additional improvement of around 0.01 in the AUC. The results for ICU admission and initial triage priority were similar with significant improvements by addition of ProADM and only minimal additional improvement with the multimarker panel.

**Table 3:** Prediction of adverse outcome of clinical models, biomarkers and combinations

	Mortality		ICU admission		High treatment priority	
	AUC (95 % CI)	*p* value	AUC (95 % CI)	*p* value	AUC (95 % CI)	*p* value
Models including clinical information readily available at ED admission						
Initial clinical model	0.79 (0.77, 0.81)	-	0.70 (0.67, 0.72)	-	0.71 (0.70, 0.72)	-
Initial clinical model plus ProADM	0.84 (0.83, 0.86)	<0.001	0.73 (0.70, 0.75)	<0.001	0.74 (0.73, 0.76)	<0.001
Initial clinical model plus multimarker	0.85 (0.83, 0.87)	<0.001	0.75 (0.73, 0.77)	<0.001	0.75 (0.74, 0.76)	<0.001
Models including full clinical information available at ED discharge						
Full clinical model	0.85 (0.83, 0.87)	-	0.75 (0.72, 0.77)	-	0.73 (0.72, 0.74)	-
Full clinical model plus ProADM	0.88 (0.87, 0.90)	<0.001	0.77 (0.75, 0.79)	<0.001	0.76 (0.75, 0.77)	<0.001
Full clinical model plus multimarker	0.89 (0.88, 0.91)	<0.001	0.79 (0.77, 0.81)	<0.001	0.77 (0.75, 0.78)	<0.001
Models including information from initial triage scores						
Triage model	0.67 (0.64, 0.70)	-	0.73 (0.72, 0.74)	-	0.79 (0.77, 0.81)	-
Triage model plus ProADM	0.83 (0.81, 0.85)	<0.001	0.76 (0.75, 0.77)	<0.001	0.84 (0.83, 0.86)	<0.001
Triage model plus multimarker	0.84 (0.82, 0.86)	<0.001	0.77 (0.75, 0.78)	<0.001	0.85 (0.83, 0.87)	<0.001

Initial clinical model includes age, gender, main presenting symptom and vital signs (i.e., heart rate, respiratory rate, blood pressure, temperature, consciousness); full clinical model includes age, gender, comorbidities, main presenting symptom, main diagnosis and vital signs; multimarker model includes ProADM, procalcitonin, copeptin; *p* value refers to comparison of combined model with the clinical model

*ICU* intensive care unit, *AUC* area under the curve, *CI* confidence interval, *ED* emergency department, *ProADM* pro-adrenomedullin

Improvements in risk assessment were also found in reclassification statistics. Table 4 shows the results of net reclassification improvement (NRI) across the three risk categories and integrated discrimination improvement (IDI) for continuous results. All models were significantly improved by the addition of ProADM (reclassification tables are presented in the Appendix). Particularly, ProADM increased the predicted risk of nonsurvivors in 14, 15 and 32 % in the different models. On the other hand, ProADM lowered the predicted risk in patients not needing ICU care by 10, 4 and 23 %.

**Table 4:** Reclassification statistics for ProADM and the three endpoints

	Mortality	ICU admission	High treatment priority
Models including clinical information readily available at ED admission			
Net reclassification improvement (NRI)	0.11 (SE 0.03), *p* = 0.00004	0.06 (SE 0.02), *p* = 0.01345	0.02 (SE 0.01), *p* = 0.00572
Integrated discrimination improvement (IDI)	0.04 (SE 0.01), *p* = 0.0001	0.01 (SE 0.01), *p* = 0.0001	0.02 (SE 0.01), *p* = 0.0001
Models including full clinical information available at ED discharge			
Net reclassification improvement (NRI)	0.09 (SE 0.02), *p* = 0.0003	0.08 (SE 0.02), *p* = 0.0001	0.02 (SE 0.01), *p* = 0.0072
Integrated discrimination improvement (IDI)	0.04 (SE 0.01), *p* = 0.0001	0.01 (SE 0.01), *p* = 0.00004	0.02 (SE 0.01), *p* = 0.0001
Models including information from initial triage scores			
Net reclassification improvement (NRI)	0.23 (SE 0.03), *p* = 0.0001	0.10 (SE 0.02), *p* = 0.0001	0.06 (SE 0.01), *p* = 0.0001
Integrated discrimination improvement (IDI)	0.05 (SE 0.01), *p* = 0.0001	0.02 (SE 0.01), *p* = 0.0001	0.01 (SE 0.01), *p* = 0.0001

Initial clinical model includes age, gender, main presenting symptom and vital signs (i.e., heart rate, respiratory rate, blood pressure, temperature, consciousness); full clinical model includes age, gender, comorbidities, main presenting symptom, main diagnosis and vital signs; multimarker model includes ProADM, procalcitonin, copeptin; *p* value refers to comparison of combined model with the clinical model

*ProADM* pro-adrenomedullin, *ICU* intensive care unit, *ED* emergency department, *SE* standard error

### Subgroup analysis

To study the robustness of our analysis across patient populations, we also investigated the prognostic performance of the markers for the three different outcomes in predefined subgroup analyses according to study center (Swiss, French, US), main medical diagnosis (infectious disease, cardiovascular disease, neurological disease), main symptom at ED presentation (nonthoracic pain, thoracic pain, respiratory symptoms, worsening of general condition, fever), age (<65 years, ≥65 years) and gender. As shown in the Appendix, results were similar in the different subgroups with the best performance in the French center, in patients with thoracic and nonthoracic pain, in patients with fever and in younger patients (age <65 years).

## Discussion

Suboptimal triage causes delays in initial treatment and impaired outcome [3, 4]. A more adequate and prompt initial triage system usable in unselected, “real-life” ED patients may allow for an improved and more targeted (“personalized”) management of patients in the ED [12]. Within this large international cohort of unselected medical patients seeking ED care, we investigated the incremental usefulness of three biomarkers from distinct biological pathways for predicting short-term mortality, ICU admission and high triage priority. ProADM emerged as the most informative biomarker for predicting adverse outcomes with high discriminatory ability particularly in regard to the primary endpoint – all-cause 30-day mortality. In addition, the use of ProADM added to the overall prediction of risk based on clinical parameters available on ED admission and ED discharge, as well as triage information. The addition of all three biomarkers in a “multimarker” model showed only minimal further improvements, as evidenced by small changes in the C-statistic. These results suggest that a combined clinical and biomarker approach may help to accurately risk stratify patients at the most proximal and challenging time point of ED admission.

Risk prediction may also assist physicians in more rational decision making regarding initial site-of-care with implications for the entire hospitalization. Reducing the number of inhospital days is important not only for cost issues [37, 38]. Hospital-acquired disability is an emerging issue in health care and older, frail medical patients are at high risk for allegedly premature referral to a nursing home with consecutive depression and further deterioration of mental and physical independence [39]. This may help to identify both high-risk patients in need of urgent care and inhospital management and low-risk patients where longer waiting times have no detrimental consequences.

Our data show large improvements in risk prediction for all outcomes when ProADM is added to the clinical risk model as evidenced by significant improvement in AUCs with best performance in the initial triage model and the initial clinical model. Particularly for identification of patients at increased risk for 30-day mortality, ProADM was most helpful. Conversely, for assessment of triage priority, ProADM improved identification of low-risk subjects, which may help to “rule out” the need for immediate medical measures. Based on these results, it is tempting to hypothesize that an initial assessment including clinical and biomarker information will improve risk stratification of patients, which may translate into better patient flow and outcomes. Still, when looking at the reclassification statistics when looking at the reclassiciation statistics of the final overall clinical model, improvements were only modest across risk categories and it thus remains unclear how many patients would benefit from biomarker testing if used in clinical practice. Thus, there is need for verification of our hypothesis in an interventional trial.

Indeed, to improve hospital management of patients with lower respiratory tract infections, we have previously developed a biomarker-enhanced clinical risk score (combining the CURB65 score and ProADM) [18, 40]. The efficacy and safety of this score was recently tested in a randomized controlled trial at one of the participating hospitals [41]. Based on these studies focusing on respiratory infections, we hypothesized that combining clinical parameters and prognostic biomarkers to an established triage risk score at the very proximal time point of ED admission, also has a substantial and clinically relevant potential to improve its performance and translate into better triage of unselected medical patients on admission and during hospitalization.

While prognostic markers have been found in different patient populations, only few investigations have found improvement in care based on the incorporation of prognostic information into “real-life” management of unselected polymorbid medical patients. To our knowledge, no study has yet investigated prognostic markers for early triage, which can reduce time to treatment – a main predictor for patient outcomes. Indeed, the robustness of our observational findings in different settings, health care systems and medical patient populations in situations of diagnostic ambiguities can be seen as a strength of this study.

We are aware of several limitations. First, treatment priority as adjudicated by two independent attending physicians at ED discharge is not a “hard” endpoint and may be subject to variation due to different levels of experience of physicians. In anticipation of this limitation, we have developed guidelines to standardize adjudication based on previous research in this field [42]. We therefore used triage priority as a secondary endpoint and also focused on other more objective endpoints (i.e., mortality, ICU admission). Second, physicians and nurses were not blinded to the triage scores and thus may adapt their priority recommendation accordingly. This may overestimate the performance of the triage scoring systems. Third, we only focused on three markers based on their performance in previous research, but other markers such as lactate or C-reactive protein (CRP) may also show benefit for early patient triage [43]. Fourth, within this observational cohort, we are not able to demonstrate whether improved triage of patients translates into better management and improved outcomes; for this reason, a randomized controlled trial ultimately testing this hypothesis needs to be done. While most prognostic blood markers (including ProADM) are now commercially available within 1 to 3 hours, faster point-of-care tests are currently being developed that will enable measurement of markers within minutes, similar to a glucose measurement [44]. This will further improve bedside use of these markers in future trials. Finally, due to differences in health care systems and logistics at the ED, the cohorts were somewhat heterogeneous in the participating centers with differences in rates of adverse outcomes and outpatient treatment (e.g., outpatient rate in the Swiss 19.8 %, French: 61.6 % and US center: 0 %). However, the robust results when looking at the prognostic accuracy of markers among the different centers is reassuring and may validate the findings for these different health care settings.

## Conclusions

In conclusion, combination of clinical information at ED admission with results of blood biomarkers allows early risk stratification in individual patients. The potential of early triage to improve patient flow, treatment times, medical outcomes and costs needs to be assessed in a randomized interventional trial. In light of the current discussion about limited health care resources, such a trial will have high relevance for our health care system.

## Key messages

- This large international emergency department study found a high prognostic accuracy of initially measured biomarkers for prediction of adverse outcome and high treatment priority
- Biomarkers improved statistical models, including comprehensive clinical information as well as triage risk scores
- The best biomarker was ProADM, particularly for mortality prediction
- Biomarker-enhanced patient triage in the emergency department has the potential to improve early patient management, patient flow and maybe also reduce adverse outcomes

## Abbreviations

ATS: Australasian Triage Scale
AUC: area under the curve
CAP: community-acquired pneumonia
CI: confidence interval
COPD: chronic obstructive pulmonary disease
CRF: case report form
CRP: C-reactive protein
CTAS: Canadian Triage and Acuity Scale
ED: emergency department
ESI: Emergency Severity Index
ICU: intensive care unit
IDI: integrated discrimination improvement
IQR: interquartile range
LOS: length of stay
MTS: Manchester Triage System
NRI: net reclassification improvement
OR: odds ratio
PCT: procalcitonin
ProADM: pro-adrenomedullin
PSI: Pneumonia Severity Index
SD: standard deviation
SE: standard error
WBC: white blood cell count
